# Supplementary material for: Germline loss-of-function PAM variants are enriched in subjects with pituitary hypersecretion
Source: Front Endocrinol (Lausanne). 2023 Jun 14;14:1166076. doi: 10.3389/fendo.2023.1166076 (PMC10303134; doi:10.3389/fendo.2023.1166076)
Supplement: Supplementary file 1 [file DataSheet_1.docx]

# **Overview of Supplemental Information**

[**Overview of Supplemental Information** 1](#_Toc131172077)

[**Supplemental Figures** 2](#_Toc131172078)

[**Supplemental Tables** 19](#_Toc131172079)

[**Supplemental Materials and Methods** 30](#_Toc131172080)

# **Supplemental Figures**

**Figure S1.** **WES analysis pipeline**

Analytic pipeline detailing the SNV filtering steps applied in the WES analysis of the index cases.

**Figure S2.** **PAM mRNA and protein expression studies in pituitary cells**

(A) tSNE map showing the expression of *Pam* gene in freshly dispersed adult rat anterior pituitary cells.  E, erythrocytes, Le, leukocytes (both negative), L, lactotropes; EC, endothelial cells; S, somatotropes; G, gonadotropes, T, thyrotropes; C, corticotropes; and FSC, folliculostellate cells (all expressing *Pam*). Data are shown on Log10 scale. (C) ﻿Immunofluorescence staining for PAM (green) and GM130 (red) in non-pathological pituitary cells (NP). PAM was visualized using an antibody (JH629) raised against the linker region of PAM-1 that recognizes intact PAM-1, soluble PHM, and membrane PAL. Magnification for all images: 40×.

**Figure S3.** **Evolutionary conservation and structure-based analyses for six missense *PAM* variants**

(A) Protein sequence alignments for five *PAM* variants in which no significant change in PHM or PAL activity was observed. Based on *in silico* and *in vitro* data, four variants are considered likely non-pathogenic, while we classified p.His240Tyr as a variant of uncertain significance (VUS). The alignments were performed using Clustal Omega with default settings using the UniProt alignment tool. Conserved affected residues are given in yellow. (B) The crystal structure of rat PHMcc (PDB entry 1OPM) was used to contextualize the p.His240Tyr *PAM* variant with respect to the active site; the WT residue is shown on the left and the mutant residue on the right. The two copper ions are depicted as green spheres, while the substrate, N-acetyl-3,5-diiodotyrosylglycine, is shown in pink.

**Figure S4.** **PHM and PAL activity for three engineered PAM variants**

As described in Methods, TMT lysates prepared from transiently transfected PEAKrapid cells were assayed for PHM activity and for PAL activity. Data for the level of expression of WT PAM and each variant were determined by quantifying the FLAG-tag signal. Data for variants expressed in any single transfection were normalized to WT PAM expressed and processed at the same time. p.Thr189Ile is located in PHMcc and p.His529Arg and p.Gly796Glu in PALcc.

**Figure S5. Allele phasing analyses**

(A) A subject with sporadic acromegaly (Belgium128) harbored two missense variants in exon 16 of *PAM*: c.1654G>A (p.Gly552Arg) and c.1688A>G (p.Asp563Gly), indicated by black solid arrows. To determine the phasing of these closely located SNVs, exon 16 was PCR-amplified from his germline DNA and the PCR product cloned into the TOPO-TA vector. Transformed bacterial colonies were screened for the presence of the variants by PCR and Sanger sequencing. Two colonies showing a different variant each are shown, confirming the subject is a compound heterozygote. MUT, mutant; WT, wild-type. (B) A subject with sporadic gigantism harbored two missense variants – c.718C>T (p.His240Tyr) and c.2276T>C (p.Phe759Ser) – located 60 kb apart (exons 9 and 20, respectively). To determine the phasing of these distantly located SNVs, we employed Single Molecule, Real Time (SMRT) Sequencing technology and a Sequel sequencer (Pacific Biosciences, Menlo Park, CA). The reads were split into the two alleles based on the heterozygous mutation walking. The coverage was continuous across the capture region with low-points of about 15x fold in either allele. The top track is the “phase 0” allele and the bottom track the “phase 1” allele. The c.718C>T variant was assigned to the phase 1 allele, while the c.2276T>C variant was assigned to the phase 0 allele, confirming that the subject is a compound heterozygote.

**Figure S6. Predicted splicing transcripts and introduced frame shift for the c.2332-2A>T variant**

(A) Diagram depicting the location of the c.2332-2A>T variant in intron 20 of *PAM* (red star) and the expected splicing events generating a normal transcript (WT) and an abnormal one lacking exon 21 (MUT). The location of the primers used for the RT-PCR analysis from blood-extracted RNA is given by the black solid arrows. MUT, mutant; WT, wild-type. (B) Protein sequence alignment for the encoded p.His778fs variant that abolishes PAL activity and reduces PHM activity (Table 3). The alignment was performed using Clustal Omega with default settings using the UniProt alignment tool. The affected residue is given in yellow when conserved. The beginning of the predicted frame shift is visible in the last row and the newly introduced amino acids are given in red.

**Figure S7. Location and *in silico* predictions for six *PAM* variants located in regulatory regions**

﻿﻿(A) Schematic representation of the *PAM* gene (GenBank: NM_000919.3, PAM-1, 25 exons) and encoded protein (NP_000910.2, 974 amino acids), including functional domains. Six functionally tested SNVs located in the promoter region and 5’UTR are shown in the zoom in panel. Variants shown to reduce PAM transcription are given in yellow, while those without an identified effect are given in white. CD, cytosolic domain; CDS, coding sequence; PAL, ﻿peptidyl-α-hydroxyglycine α-amidating lyase; PHM, peptidylglycine α-hydroxylating monooxygenase; TMD, transmembrane domain; UTR, untranslated region. (B) Genomatix prediction depicting the transcription factor (TF) binding sites perturbed by the six variants. The MatInspector v3.1 tool of Genomatix (Matrix Library 11.0) was used with a core similarity threshold of 0.75 and an optimized matrix similarity threshold to search for the presence of TF binding sites. We limited our search to vertebrate general core promoter elements and the + strand. WT, wild-type.

**Figure S8. Functional study for the c.*1455C>T 3’UTR variant**

(A) Cartoon depicting the experimental setup and the expected outcome based on the *in silico* predictions. The image was adapted from the siCHECK^TM^ Vectors Technical Bulletin (TB329, Promega). The WT and mutant 3’UTRs of *PAM* were PCR-amplified from human genomic DNA and cloned into the psiCHECK-2 vector, downstream of the *Renilla* Luciferase CDS. The constructs were transiently transfected into HEK-293 AD cells together with a *Firefly* reporter for normalization and each of the four miRNAs mimics predicted to bind the mutated 3’UTR. *Firefly* and *Renilla* activities were then measured. MUT: mutant; UTR, untranslated region; WT, wild-type. (B) miRNAs were initially transfected at 30 nM (n = 2 biological experiments each with three technical replicates, upper panel); the concentration of miR-556 was subsequently increased to 50 nM (n = 2 biological experiments each with three technical replicates, lower panel). This analysis failed to find a significant decrease in luciferase activity, suggesting that the mutant 3’UTR of *PAM* is not bound by the predicted miRNAs.

**Figure S9. Loss of heterozygosity (LOH) analysis for *PAM* in the pituitary adenoma of the individual Belgium197 compound heterozygote for the p.[Gly552Arg];p.[Asp563Gly] variants**

The DNA was extracted from unstained sections and LOH was investigated by Sanger sequencing. The primers used for the PCR amplification of exon 16, which harbors both variants observed in the germline DNA (Figure 2A), are reported in Table S1. The black solid arrows indicate the affected nucleotides. HET, heterozygous; WT, wild-type.

**Figure S10. Rare missense variants identified in the UKBB as significantly associated to diagnoses of tumoral/hyperfunctioning pituitary gland**

(A) Protein sequence alignments were performed using Clustal Omega with default settings using the UniProt alignment tool. Conserved affected residues are given in yellow. Only the variants located in PHMcc and PALcc are shown. (B) The crystal structure of rat PALcc (PDB entry 3FW0) was used to contextualize the missense variants; the WT residue is shown on the left and the mutant residue on the right. ﻿PAL folds as a β-propeller, with six blades (numbered 1 to 6) positioned around a central cavity. The affected residues are visualized as sticks and highlighted in dark orange, along the ribbon visualization of WT rat PALcc in grey. C, C-terminus; N, N-terminus.

**Figure S11. Bioinformatic and *in vitro* analyses for the intronic c.2746+3A>T (p.(Ala897_Arg915del))**

***PAM* variant identified in the UKBB**

(A) The Alamut Visual 2.9 software was employed for splicing assessment. The variant was predicted to reduce the usage of the natural splice donor site in intron 24 by all four splicing tools. (B) The variant was functionally tested with a minigene splicing assay. *PAM* exon 24 and flanking intronic sequences were cloned into the pSPL3 vector. The full-length insert is reported in the Supplemental Methods. Then, the c.2746+3A>T variant was introduced by site-directed mutagenesis. The WT and mutant vectors were transfected into HEK-293 AD cells alongside an eGFP plasmid. 24h post-transfection, RNA was extracted and RT-PCR was performed using both pSPL3-specific (SD6 + SA2) and eGFP primers. Two replicates are shown for each condition. In both the WT and mutant constructs, the two upper bands represent non-specific splicing (white arrow), while the lower band in WT represents correct splicing. The variant causes a reduction in the amount of correctly spliced transcripts, favoring exon skipping (260 bp band, same as in MOCK). Densitometric quantification of both bands (enclosed by white dotted squares) was evaluated using the ImageJ software. The results were normalized on eGFP expression. All bands were confirmed by sequencing. MOCK, cDNA from empty vector-transfected cells consisting of a 260 bp band made up of fragments of pSPL3 exons; MW, molecular weight marker; UNTR, untransfected; WT, wild-type; --, negative control (RT-PCR without cDNA).

**Figure S12. Splicing and mRNA expression studies for selected *PAM* variants found in our cohort**

(A) ﻿﻿Schematic representation of the *PAM* gene (GenBank: NM_000919.3, PAM-1, 25 exons) and encoded protein (NP_000910.2, 974 amino acids), including functional domains. Eleven functionally tested SNVs located along the gene are shown in the zoom in panels. CD, cytosolic domain; CDS, coding sequence; PAL, ﻿peptidyl-α-hydroxyglycine α-amidating lyase; PHM, peptidylglycine α-hydroxylating monooxygenase; TMD, transmembrane domain; UTR, untranslated region. (B) Nine *PAM* SNVs were evaluated for their effect on splicing using a minigene assay. The variants were cloned into the pSPL3 vector and transfected into HEK-293 AD cells. The full-length inserts are reported in the Supplemental Methods. 24h post-transfection, RNA was extracted and RT-PCR was performed using vector-specific primers (SD6 + SA2). The three synonymous variants are given in blue, the missense variant is given in green, and the remaining five intronic variants are given in black. None of these variants affected splicing. The effect on splicing of the missense c.92T>A (p.Phe31Tyr) and the synonymous c.2145C>A (p.Thr715=) SNVs was also determined in lymphocytes-extracted RNA, which confirmed the minigene outcomes (not shown). The missense c.92T>A (p.Phe31Tyr) in exon 2 was tested because it is located 3 bp downstream of a splice acceptor site and was predicted to activate a cryptic splice donor site. In all exon 2 constructs, the upper band represents normal splicing, while the lower band represents non-specific splicing. All bands were confirmed by sequencing. MW, molecular weight marker; MOCK, cDNA from empty vector-transfected cells consisting of a 260 bp band made up of fragments of pSPL3 exons; WT, wild-type; --, negative control (RT-PCR without cDNA). The lines separating c.1614-32G>A and negative control from exons 18-20 constructs indicate that data for intervening samples were removed. (C) The c.195G>T (p.Gly65=) variant was subsequently studied by RT-qPCR to assess whether it affects *PAM* mRNA expression. HEK-293 AD cells were transfected with WT, mutant, and WT plus mutant (to mimic a heterozygous state) plasmids. 24h post-transfection, RNA was extracted and RT-qPCR was performed using a TaqMan assay designed to specifically amplify the correctly spliced human *PAM* exon 2 transcript. The variant did not affect *PAM* mRNA expression. WT, wild-type.

**Figure S13. Effect of PAM silencing on pituitary cell proliferation**

(A) Rat pituitary tumor GH- and prolactin-secreting cells (GH3) were transiently transfected with *Pam* siRNA. Non-targeting (NT) siRNA was used as negative control. Successful silencing of PAM expression was verified in cell lysates by Western blot analysis using an anti-PAM antibody (Ab JH629, 1:1000). Beta Actin (1:2000) was used as loading control. (B) 96 h post-transfection, cell proliferation was measured by BrdU incorporation into newly synthesized DNA. ﻿OD was determined at 450 nm.

# **Supplemental Tables**

**Table S2. Primers used in this study**

| **Application** | **Primer name** | **Sequence (5’-3’)** | **Product size (bp)** |
| --- | --- | --- | --- |
| Sanger sequencing  screening | PAM_prom1_F | GGGGCAGCTAAGATTCTCCC | 732 |
|  | PAM_prom1_R | GCAAATCCCAGCTGAGGTTC |  |
|  | PAM_prom2_F | GGCTGTGAGGGATAAAGGGG | 699 |
|  | PAM_prom2_R | ACAAGGAGAACTAGCAGGCT |  |
|  | PAM_ex1_F | GCCATGAAGTAGCGGCTG | 233 |
|  | PAM_ex1_R | CAACAGCATGCACAGAAAGC |  |
|  | PAM_ex2_F | CCTTTCTTTCCCCTTCTCATGT | 285 |
|  | PAM_ex2_R | AGTTCCTACAGCTGTAAATGCA |  |
|  | PAM_ex3_F | GCCACTCAGCCCTCAGAA | 239 |
|  | PAM_ex3_R | ACTGCCCTCCATTAAACTGC |  |
|  | PAM_ex4_F | TGTATTTTATGACTTTGGTGCACAG | 229 |
|  | PAM_ex4_R | GCACAGATACACTTTGCACTTG |  |
|  | PAM_ex5_F | CCCATCACCTCCCACCATG | 270 |
|  | PAM_ex5_R | CAGAACACTGTCAACACTGGT |  |
|  | PAM_ex6_F | ACTCTGGTTCTGAAGTGGGA | 342 |
|  | PAM_ex6_R | TGCTTAGTTCCAACACTTCAAAA |  |
|  | PAM_ex7_F | AGTATGTCTGCAGGAAAGTCAA | 392 |
|  | PAM_ex7_R | CTTCCCACTCTAAGTTCGCA |  |
|  | PAM_ex8_F | AGGAACCATTTAGAGTCTGCCT | 490 |
|  | PAM_ex8_R | ACACAAGGGCCTCAATTCCT |  |
|  | PAM_ex9_F | CCTCAGTCCCTAGCATCTTGT | 246 |
|  | PAM_ex9_R | TTTATGGCACCAGGAAGCAC |  |
|  | PAM_ex10_F | ACTCAGTCTTTGCAGCTCTG | 329 |
|  | PAM_ex10_R | AGCTCAGTTATACAGTTAAGGCA |  |
|  | PAM_ex11_F | ACCTCAACACAGTCAAACATGT | 249 |
|  | PAM_ex11_R | GCTGTCACCTGTAATCATGCC |  |
|  | PAM_ex12_F | TGGCAGATCTAAGGGCTTCA | 386 |
|  | PAM_ex12_R | TGTGACTGGTAGAAGAAGGGA |  |
|  | PAM_ex13_F | ACGGTAATAAGCACAAGACCC | 300 |
|  | PAM_ex13_R | TGAGGCAGATACAGAGAGACA |  |
|  | PAM_ex14_F | CCTATGCTTAAAAGTCTGAGTGC | 519 |
|  | PAM_ex14_R | TGCTAACGTGGATTGTAGCATTT |  |
|  | PAM_ex15_F | GAGGCAATTCGACCTTTCCC | 365 |
|  | PAM_ex15_R | ACACAAGACTTCCTCTCTGCT |  |
|  | PAM_ex16_F | TGTGAATGGTCTGCATTTCTCA | 291 |
|  | PAM_ex16_R | GTGCAGTTATACAAAACACTCGA |  |
|  | PAM_ex17_F | CTGTCACATATGCCTTGAGAGT | 208 |
|  | PAM_ex17_R | ACCCATACAACTCCAGAGCT |  |
|  | PAM_ex18_F | ACCATGAAAAGTAGGTAAGGCT | 297 |
|  | PAM_ex18_R | ACTGGCTAAGAATTACAACTGGA |  |
|  | PAM_ex19_F | AGAGAGTCAAACTTTGGGTGAT | 497 |
|  | PAM_ex19_R | TTTTAACCCCTTACAAACAGGTG |  |
|  | PAM_ex20_F | TTGTAGGGACCAGGTTGACC | 372 |
|  | PAM_ex20_R | ACACTCCCATCAACCTTGCT |  |
|  | PAM_ex21_F | TGTTGTGGGCTTTGCTTTGT | 424 |
|  | PAM_ex21_R | AGACAGTACTTCAGGCATCAAGA |  |
|  | PAM_ex22_F | TCCAGGATTCATGGACCACC | 286 |
|  | PAM_ex22_R | AGAATTCAACACAGCCAGACT |  |
|  | PAM_ex23_F | ATTTGACTGGGTAGGGGTGG | 366 |
|  | PAM_ex23_R | TCCCAATCAAACTCCTGAGGA |  |
|  | PAM_ex24_F | GAGCCAGGCCTTGCTTTATC | 223 |
|  | PAM_ex24_R | TGTGCTTGTTGACTTTGAACCA |  |
|  | PAM_ex25_F | CTTTCCCATCCCCACCTTCT | 397 |
|  | PAM_ex25_R | GTCCCACACAGACTAGTACAG |  |
| LOH | PAM_ex16LOH_F | TGTGAATGGTCTGCATTTCTCA | 173 |
|  | PAM_ex16LOH_R | GGAGTACTGCAGCATTATTTGG |  |
| Mutagenesis | PAM_V27I_F | CCACCTCTTAAAGATAGAAAGTGGGCTTCGGAAAG |  |
|  | PAM_V27I_R | CTTTCCGAAGCCCACTTTCTATCTTTAAGAGGTGG |  |
|  | PAM_F31Y_F | AAATGGTCTGGTAGTTTCTTTATACCTCTTAAAGACAGAAAGTGG |  |
|  | PAM_F31Y_R | CCACTTTCTGTCTTTAAGAGGTATAAAGAAACTACCAGACCATTT |  |
|  | PAM_V49L_F | TGAATCAATAGGAACTAGGGGTCTGGTGGTACCA |  |
|  | PAM_V49L_R | TGGTACCACCAGACCCCTAGTTCCTATTGATTCA |  |
|  | PAM_R80Q_F | CCTCATCCACTGGTATTTGCATAGACATGCAGAAG |  |
|  | PAM_R80Q_R | CTTCTGCATGTCTATGCAAATACCAGTGGATGAGG |  |
|  | PAM_T189I_F | GTGGCAGACGTATGAGGTGTAAGGACACACCAG |  |
|  | PAM_T189I_R | CTGGTGTGTCCTTACACCTCATACGTCTGCCAC |  |
|  | PAM_H240Y_F | CACTTACTACCTTACCTAAATAGTGAGTGTGAACTCTATAGGC |  |
|  | PAM_H240Y_R | GCCTATAGAGTTCACACTCACTATTTAGGTAAGGTAGTAAGTG |  |
|  | PAM_V244A_F | TTACTCTGTATCCACTTACTGCCTTACCTAAATGGTGAGTG |  |
|  | PAM_V244A_R | CACTCACCATTTAGGTAAGGCAGTAAGTGGATACAGAGTAA |  |
|  | PAM_P412L_F | CTCTGATTCTGCCTTTTCTGTAAGATTATATTTGTGCACATGAAC |  |
|  | PAM_P412L_R | GTTCATGTGCACAAATATAATCTTACAGAAAAGGCAGAATCAGAG |  |
|  | PAM_I452V_F | TTCTGTCTCTGACAAGAACAGCATTACCCCTCTCATG |  |
|  | PAM_I452V_R | CATGAGAGGGGTAATGCTGTTCTTGTCAGAGACAGAA |  |
|  | PAM_E491D_F | GGAAATCTCCTGTGTGGTCTGGTTCCCAGGTGC |  |
|  | PAM_E491D_R | GCACCTGGGAACCAGACCACACAGGAGATTTCC |  |
|  | PAM_S539W_F | CAAACTTGCTGTCAAACCAGTTTCCATCCCAGACA |  |
|  | PAM_S539W_R | TGTCTGGGATGGAAACTGGTTTGACAGCAAGTTTG |  |
|  | PAM_G552R_F | GTGTCTTCTTCAATTGGTCTGAGTCCTATTTGCTGGTAA |  |
|  | PAM_G552R_R | TTACCAGCAAATAGGACTCAGACCAATTGAAGAAGACAC |  |
|  | PAM_D563G_F | GTACTGCAGCATTATTTGGACCTATGACAAGAATAGTGTCTTCTTC |  |
|  | PAM_D563G_R | GAAGAAGACACTATTCTTGTCATAGGTCCAAATAATGCTGCAGTAC |  |
|  | PAM_G641R_F | GGCTCCAGTGCGTGGATCCACAGCC |  |
|  | PAM_G641R_R | GGCTGTGGATCCACGCACTGGAGCC |  |
|  | PAM_R703Q_F | GGATCCGACCATTTTCCTGGTCTGCCACACATAAT |  |
|  | PAM_R703Q_R | ATTATGTGTGGCAGACCAGGAAAATGGTCGGATCC |  |
|  | PAM_F759S_F | GAACCTGTACAAGGATCTGTGATGAACTTTTCC |  |
|  | PAM_F759S_R | GGAAAAGTTCATCACAGATCCTTGTACAGGTTC |  |
|  | PAM_P51=_F | CAAAATCTGATGAATCAATGGGAACTACGGGTCTGGTG |  |
|  | PAM_P51=_R | CACCAGACCCGTAGTTCCCATTGATTCATCAGATTTTG |  |
|  | PAM_G65=_F | GACTGTTTAGGTGTAACACCAGGCATGCGAATATC |  |
|  | PAM_G65=_R | GATATTCGCATGCCTGGTGTTACACCTAAACAGTC |  |
|  | PAM_T715=_F | CTTAATCTCTCTCACAAATTCTTTTGTGTCAGTTTTAAAACACTGGATC |  |
|  | PAM_T715=_R | GATCCAGTGTTTTAAAACTGACACAAAAGAATTTGTGAGAGAGATTAAG |  |
|  | PAM_c.92T>A_F | AAAATGGTCTGGTAGTTTCTTTATACCTTAAAAAAGAGGGCAATTTC |  |
|  | PAM_c.92T>A_R | GAAATTGCCCTCTTTTTTAAGGTATAAAGAAACTACCAGACCATTTT |  |
|  | PAM_c.443-79G>T_F | GTTAATCTCAAATTGCTGTTCTATGAAATCCAAAGGGAAATAACATATAACATAT |  |
|  | PAM_c.443-79G>T_R | ATATGTTATATGTTATTTCCCTTTGGATTTCATAGAACAGCAATTTGAGATTAAC |  |
|  | PAM_c.443-5T>C_F | AACTCTGAATCCAACACCTAAGGATTTACAAAAAAGTTAATATATTAGAAATGAGC |  |
|  | PAM_c.443-5T>C_R | GCTCATTTCTAATATATTAACTTTTTTGTAAATCCTTAGGTGTTGGATTCAGAGTT |  |
|  | PAM_c.1614-32G>A_F | GCATTAAATAGGACATGAAATAATCATAAATTCCAATGAAATTGAGAAATGCAGAC |  |
|  | PAM_c.1614-32G>A_R | GTCTGCATTTCTCAATTTCATTGGAATTTATGATTATTTCATGTCCTATTTAATGC |  |
|  | PAM_c.1804-5A>T_F | CAGTTTGAACACCTTTATAAAAAGGAACAAAAGCCTTACCTACTTT |  |
|  | PAM_c.1804-5A>T_R | AAAGTAGGTAAGGCTTTTGTTCCTTTTTATAAAGGTGTTCAAACTG |  |
|  | PAM_c.2331+76A>C_F | TCTTAAAAGTTTTTCTAAAGCTATTCAAGGACAGATTGAATGCCAAGATTTATATTTTA |  |
|  | PAM_c.2331+76A>C_R | TAAAATATAAATCTTGGCATTCAATCTGTCCTTGAATAGCTTTAGAAAAACTTTTAAGA |  |
|  | PAM_c.2332-2A>T_F | CATGAGGCATATCAAAGTGCAGCCAAAATAAGAAGCTACAC |  |
|  | PAM_c.2332-2A>T_R | GTGTAGCTTCTTATTTTGGCTGCACTTTGATATGCCTCATG |  |
|  | PAM_c.2746+3A>T_F | AGGTCATAGGCAAACCTCTAAATCTTCCCAGTACTC |  |
|  | PAM_c.2746+3A>T_R | GAGTACTGGGAAGATTTAGAGGTTTGCCTATGACCT |  |
|  | PAM_c.-607A>G_F | GCTCTTCCTCCCTCGGACCCACTCCTG |  |
|  | PAM_c.-607A>G_R | CAGGAGTGGGTCCGAGGGAGGAAGAGC |  |
|  | PAM_c.-550G>T_F | AAGATTTCAGGAACACCCACGGCGCAGCC |  |
|  | PAM_c.-550G>T_R | GGCTGCGCCGTGGGTGTTCCTGAAATCTT |  |
|  | PAM_c.-361G>A_F | AAACCCGCGTCAGTTATCATTCAGAACCTGCAAAAATAG |  |
|  | PAM_c.-361G>A_R | CTATTTTTGCAGGTTCTGAATGATAACTGACGCGGGTTT |  |
|  | PAM_c.-133T>C_F | CTGCGGCGGCCGCACACCATCCG |  |
|  | PAM_c.-133T>C_R | CGGATGGTGTGCGGCCGCCGCAG |  |
|  | PAM_c.-109G>C_F | GCCCGGGGACGGCGGGCGTCC |  |
|  | PAM_c.-109G>C_R | GGACGCCCGCCGTCCCCGGGC |  |
|  | PAM_c.-35C>G_F | GGGAGAGGACCCGGCAGCGCAGC |  |
|  | PAM_c.-35C>G_R | GCTGCGCTGCCGGGTCCTCTCCC |  |
| Cloning | PAM_ex2_pSPL3_F | GGGATCACCAGAATTCCAATCATCTTTAAGGTCATAGAATT | 321 |
|  | PAM_ex2_pSPL3_R | TCGAGCTCCAGAATTCACGTTTAAAGAAGTTCCTACAGCTG |  |
|  | PAM_ex6_pSPL3_F | GGGATCACCAGAATTCTTATCAGTCAATGGTATATTTCTGT | 384 |
|  | PAM_ex6_pSPL3_R | TCGAGCTCCAGAATTCAGTTCCAAATTAAAGGAAACAAAAG |  |
|  | PAM_ex16_pSPL3_F | GGGATCACCAGAATTCTTGAGCTCTTTTGATTTTGTATTTA | 317 |
|  | PAM_ex16_pSPL3_R | TCGAGCTCCAGAATTCAGTGCAGTTATACAAAACACTCGAA |  |
|  | PAM_ex18_pSPL3_F | GGGATCACCAGAATTCGCTTCTACTCCAATTGCTTG | 411 |
|  | PAM_ex18_pSPL3_R | TCGAGCTCCAGAATTCGGACAATTACAGCTTAAAGAA |  |
|  | PAM_ex19_pSPL3_F | GGGATCACCAGAATTCCATTTAATGGTTTACTATCATGAAT | 401 |
|  | PAM_ex19_pSPL3_R | TCGAGCTCCAGAATTCTAGCAAAGAAAATTTATAAAAAGAG |  |
|  | PAM_ex20_pSPL3_F | GGGATCACCAGAATTCAGACACTAAGTATTTTATATTCTAC | 416 |
|  | PAM_ex20_pSPL3_R | TCGAGCTCCAGAATTCAAATAGAACACTCCCATCAACCTTG |  |
|  | PAM_ex21_pSPL3_F | GGGATCACCAGAATTCTTTGTTGTTTTGTTGTGGGCTTTGC | 300 |
|  | PAM_ex21_pSPL3_R | TCGAGCTCCAGAATTCATCAGTAAAAAGATAAGGAAATGCA |  |
|  | PAM_ex24_pSPL3_F | GGCAGGAGTCAAACCCAGC | 554 |
|  | PAM_ex24_pSPL3_R | GACATGCCAACAGGAGTGACAG |  |
|  | PAM_3’UTR_psiCHECK2_F | TAGGCGATCGCTCGAGAAACCAAGCTTTGATTTAGATTGAG | 2040 |
|  | PAM_3’UTR_psiCHECK2_R | TTGCGGCCAGCGGCCGCTTTCCCTTTGCTTTTTTAATAAGTC |  |
|  | PAM_CDS_pCMV-FLAG-C_F | AGATCTGCCGCCGCGATCGCATGGCTGGCCGCGTCCCTAGCCTGC | 2922 |
|  | PAM_CDS_pCMV-FLAG-C_R | GCGGCCGCGTACGCGTGGAGGAGGAAGGTGCGAGCGCAGGC |  |
| Expression constructs sequencing | PAM_seq_F1 | AGGAAGCCTTCGTGATTGACT |  |
|  | PAM_seq_F2 | GGACACTGATTGGACGGCA |  |
|  | PAM_seq_F3 | GACCTGGTAGCTGAGATTGCA |  |
|  | PAM_seq_F4 | TGGGAATTATTGGGTCACAGAC |  |
|  | PAM_seq_F5 | GGTTATTCCGGTGGTTGTCC |  |
|  | PAM_seq_R1 | GGAACTACGGGTCTGGTGGT |  |
|  | PAM_seq_R2 | CTTCTCGTTTTGGCTGCTGT |  |
|  | PAM_seq_R3 | TGGTCCGAGTCCTATTTGCTG |  |
|  | PAM_seq_R4 | TTTCCTGGACCTCAATGCCA |  |
| qPCR | PAM_ex2_norm splic_F | GATTCATCAGATTTTGCATTGGAT | 79 |
|  | PAM_ex2_norm splic_R | GGGTCCCCTCGGGAGAT |  |
|  | PAM_ex2_norm splic_probe | TTACACCTAAACAGACCTGG |  |
| RT-PCR | eGFP_F | ATCATGGCCGACAAGCAGAA | 187 |
|  | eGFP_R | TCTCGTTGGGGTCTTTGCTC |  |
| PCR splicing to delete exon 21 | PAM_flank-ex21_F | GACCTGGTAGCTGAGATTGCA | 1086 |
|  | PAM_ex21del_R | ATCGATGTTCCAATTCTTGCGCACTGGCTT |  |
|  | PAM_ex21del_F | AAGCCAGTGCGCAAGAATTGGAACATCGAT | 63 |
|  | PAM_flank-ex21_R | TTTCCTGGACCTCAATGCCA |  |
| Minigene assay | SD6 | TCTGAGTCACCTGGACAACC |  |
|  | SA2 | ATCTCAGTGGTATTTGTGAGC |  |

**Table S3. Candidate variants in index family**

| **Gene** | **Chromosome** | **DNA**  **change** | **Protein change** | **RefSeq** | **SNP ID** | **Global Control MAF (%)** | ***In silico* prediction** |
| --- | --- | --- | --- | --- | --- | --- | --- |
| *ITGB5* | 3 | c.1262C>T | p.Thr421Met | NM_002213.5 | rs200415084 | 0.0039 | 7, 9, 2 |
| *THBS4* | 5 | c.1771C>T | p.Arg591Trp | NM_003248.6 | rs148589675 | 0.0368 | 2, 6, 9 |
| *AVL9* | 7 | c.243A>C | p.Arg81Ser | NM_015060.3 | rs142055726 | 0.1156 | 0, 7, 10 |
| *CEP350* | 1 | c.6130A>C | p.Thr2044Pro | NM_014810.5 | rs56173179 | 0.3654 | 2, 11, 3 |
| *ANKRD36C* | 2 | c.3910A>T | p.Asn1304Tyr | NM_001310154.3 | rs768682466 | 0.2027 | 0, 4, 9 |
| ***PAM*** | **5** | **c.2108G>A** | **p.Arg703Gln** | **NM_000919.3** | **rs761130902** | **0.0013** | **10, 9, 0** |
| *TRMT11* | 6 | c.869A>G | p.Asp290Gly | NM_001031712.2 | rs781238788 | 0.0007 | 10, 6, 2 |
| *PXDNL* | 8 | c.3352C>G | p.Leu1118Val | NM_144651.5 | rs145542518 | 0.3009 | 0, 4, 13 |
| *UHRF2* | 9 | c.869C>G | p.Ser290Cys | NM_152896.3 | rs373417432 | 0.0059 | 1, 8, 8 |
| *ZFYVE19* | 15 | c.1192C>T | p.Arg398Cys | NM_001077268.2 | rs72735636 | 0.1887 | 2, 1, 14 |
| *ZNF799* | 19 | c.1481A>G | p.His494Arg | NM_001080821.3 | rs151038912 | 0.1275 | 4, 5, 7 |
| *ABCB6* | 2 | c.1762G>A | p.Gly588Ser | NM_005689.4 | rs145526996 | 0.4192 | 5, 12, 0 |
| *ARAP2* | 4 | c.2050G>A | p.Asp684Asn | NM_015230.3 | rs200497411 | 0.0112 | 2, 5, 10 |
| *RPS3A* | 4 | c.598G>C | p.Ala200Pro | NM_001006.5 | rs781597345 | 0.0007 | 4, 9, 4 |
| *CCNO* | 5 | c.134G>T | p.Pro45His | NM_021147.5 | rs139606873 | 0.1459 | 0, 4, 13 |
| *SEPTIN14* | 7 | c.796T>C | p.Tyr266His | NM_207366.2 | rs368110863 | 0.0158 | 8, 5, 4 |
| *FBXO24* | 7 | c.17T>A | p.Val6Asp | NM_033506.3 | rs148663467 | 0.0269 | 0, 3, 14 |
| *CFTR* | 7 | c.2168G>T | p.Gly723Val | NM_000492.4 | rs200531709 | 0.0013 | 2, 10, 6 |
| *OR2A14* | 7 | c.736G>A | p.Val246Met | NM_001001659.1 | rs201108825 | 0.0177 | 1, 4, 12 |
| *CD163L1* | 12 | c.3163G>A | p.Gly1055Ser | NM_174941.5 | rs36206713 | 0.6261 | 1, 7, 9 |
| *CFAP251* | 12 | c.2578C>T | p.Arg860Cys | NM_001178003.1 | rs146415200 | 0.5433 | 2, 4, 10 |
| *SBNO1* | 12 | c.614A>G | p.Asn205Ser | NM_018183.3 | rs199607415 | 0.1925 | 0, 8, 8 |
| *ATP12A* | 13 | c.1883G>A | p.Arg628Gln | NM_001185085.2 | rs770792516 | 0.0013 | 5, 9, 4 |
| *HIF1A* | 14 | c.148G>C | p.Val50Leu | NM_181054.2 | rs61755705 | 0.2135 | 0, 13, 4 |
| *NRDE2* | 14 | c.1519G>A | p.Val507Met | NM_017970.3 | rs79341977 | 0.4136 | 2, 9, 5 |
| *GATM* | 15 | c.67G>A | p.Arg23Trp | NM_001482.3 | rs1240389133 | 0.0013 | 0, 10, 9 |
| *LIPC* | 15 | c.1214C>T | p.Thr405Met | NM_000236.2 | rs113298164 | 0.2839 | 5, 7, 5 |

Twenty-seven nonsynonymous variants passed the variant prioritization process in the individuals screened by WES. In light yellow are given variants that fully segregated with the phenotype in all three generations, in light green are given variants that fully segregated with the phenotype only in generation II, and in light orange are given variants that fully segregated with the phenotype only in generation III. The *PAM* variant is highlighted in bold. MAFs were retrieved from the gnomAD database ver. 3.1.2. For *in silico* predictions, the computational verdict was based on the combined outputs of 19 software available in Varsome: the first number represents the pathogenic verdicts, the second number the uncertain verdicts, and the third number the benign verdicts.

MAF, minor allele frequency; n.a., not available; SA, splice acceptor site; VUS, variant of uncertain significance.

**Table S4. *PAM* SNVs detected in our cohort of individuals with pituitary adenomas but not functionally tested**

| **DNA change** | **Protein change** | **SNP ID** | **Location in gene** | **Location in protein** | **Global Control MAF (%)** | **Highest Control Population MAF (%)** | **# of homozygotes in gnomAD** | ***In silico* prediction** |
| --- | --- | --- | --- | --- | --- | --- | --- | --- |
| c.-1069delT | n.a. | rs3834823 | promoter | n.a. | 9.088 | 26.27 (African) | 1560 | benign |
| c.-1009G>T | n.a. | rs1231126774 | promoter | n.a. | 0.0007 | 0.0024 (African) | 0 | benign |
| c.-310C>G | n.a. | rs249496 | 5’UTR (exon 1) | n.a. | 51.34 | 72.21 (African) | 21840 | benign |
| c.89+88G>A | n.a. | rs73192734 | intron 1 | n.a. | 8.920 | 25.33 (African) | 1486 | benign |
| c.211-78A>G | n.a. | rs73175443 | intron 2 | n.a. | 3.012 | 10.40 (African) | 222 | benign |
| c.269-185T>C | n.a. | rs149240405 | intron 3 | n.a. | 0.4472 | 2.083 (Amish) | 5 | benign |
| c.269-92A>G | n.a. | rs6876278 | intron 3 | n.a. | 26.94 | 43.31 (East Asian) | 5911 | benign |
| c.269-51G>T | n.a. | rs112746759 | intron 3 | n.a. | 1.352 | 2.115 (European) | 18 | benign |
| c.443-121A>G | n.a. | rs542812907 | intron 5 | n.a. | 0.0861 | 3.838 (Amish) | 1 (Amish) | benign |
| c.527-69T>A | n.a. | rs3776873 | intron 6 | n.a. | 28.75 | 43.13 (East Asian) | 6539 | benign |
| c.576-145G>A | n.a. | rs143408776 | intron 7 | n.a. | 0.6669 | 1.241 (European) | 8 (European) | benign |
| c.576-68G>A | n.a. | rs140557297 | intron 7 | n.a. | 1.099 | 1.701 (European) | 4 | benign |
| c.576-14A>G | n.a. | rs182339803 | intron 7 | n.a. | 0.6395 | 2.038 (African) | 10 | benign |
| c.906-75G>T | n.a. | rs2195272 | intron 11 | n.a. | 28.65 | 43.10 (East Asian) | 6510 | benign |
| c.1483+26G>A | n.a. | rs3733939 | intron 13 | n.a. | 40.91 | 54.11 (African) | 13363 | benign |
| c.1484-98A>G | n.a. | rs12656131 | intron 14 | n.a. | 31.17 | 43.81 (East Asian) | 7542 | benign |
| c.1614-125A>G | n.a. | rs1451051658 | intron 15 | n.a. | n.a. | n.a. | n.a. | benign |
| c.2014+27G>A | n.a. | rs114859298 | intron 18 | n.a. | 0.1295 | 4.434 (African) | 43 | benign |
| c.2015-171T>C | n.a. | rs45518833 | intron 18 | n.a. | 25.14 | 43.66 (East Asian) | 5278 | benign |
| c.2215+139G>A | n.a. | rs73179842 | intron 19 | n.a. | 3.573 | 12.60 (African) | 358 | benign |
| c.2307A>C | p.Ile769= | rs2230457 | exon 20 | PAL | 25.01 | 43.65 (East Asian) | 5256 | benign |
| c.2431+147G>A | n.a. | rs11960579 | intron 21 | n.a. | 3.875 | 13.55 (African) | 385 | benign |
| c.2431+154T>A | n.a. | rs30719 | intron 21 | n.a. | 3.017 | 7.723 (South Asian) | 76 | benign |
| c.2432-142A>G | n.a. | rs77585944 | intron 21 | n.a. | 1.470 | 4.539 (Finnish) | 27 | benign |
| c.2432-23C>G ^†^ | n.a. | rs183956071 | intron 21 | n.a. | 0.6695 | 3.719 (Finnish) | 15 | benign |
| c.2486-125A>C | n.a. | rs79989892 | intron 22 | n.a. | 1.081 | 3.681 (African) | 22 (African) | benign |
| c.2486-88A>C | n.a. | rs17296280 | intron 22 | n.a. | 27.44 | 43.83 (Amish) | 6177 | benign |
| c.2689+46G>C | n.a. | rs41300805 | intron 23 | n.a. | 7.290 | 11.38 (Finnish) | 1532 | benign |
| c.2689+118C>T | n.a. | rs147272823 | intron 23 | n.a. | 1.313 | 9.226 (Amish) | 15 | benign |
| c.2690-28C>T | n.a. | rs26433 | intron 23 | n.a. | 64.72 | 84.54 (South Asian) | 32347 | benign |
| c.2747-49_2747-48insAT | n.a. | rs2067133 | intron 24 | n.a. | 74.80 | 86.52 (African) | 43021 | benign |
| c.*417G>A | n.a. | rs5855 | 3’UTR (exon 25) | n.a. | 68.90 | 84.65 (South Asian) | 36249 | benign |
| c.*941T>A | n.a. | rs26432 | 3’UTR (exon 25) | n.a. | 68.89 | 84.73 (South Asian) | 36237 | benign |
| c.*1025G>C | n.a. | rs26431 | 3’UTR (exon 25) | n.a. | 74.60 | 85.72 (African) | 42792 | benign |
| c.*1163G>A | n.a. | rs26430 | 3’UTR (exon 25) | n.a. | 68.90 | 84.71 (South Asian) | 36246 | benign |
| c.*1284G>A | n.a. | rs26429 | 3’UTR (exon 25) | n.a. | 68.91 | 84.69 (South Asian) | 36256 | benign |
| c.*1715C>T | n.a. | rs26428 | 3’UTR (exon 25) | n.a. | 68.90 | 84.66 (South Asian) | 36271 | benign |
| c.*1748A>C | n.a. | rs73181445 | 3’UTR (exon 25) | n.a. | 1.624 | 5.710 (African) | 63 | benign |
| c.*2014A>T | n.a. | rs26427 | 3’UTR (exon 25) | n.a. | 4.170 | 14.57 (African) | 441 | benign |

In addition to the prioritized variants, 38 other variants (10 in regulatory regions, one synonymous, and 27 intronic) were detected in our cohort. Variants were annotated using the NC_000005.9(NM_000919.3) reference sequence. MAFs were retrieved from the gnomAD database. “European” excludes the Finnish population; “African” includes African-American; “Latino” includes Admixed American. For *in silico* predictions, the computational verdict was based on the output reported in Varsome.

MAF, minor allele frequency; n.a., not applicable.

^†^The subject harbors the 5’UTR c.-361G>A *PAM* variant.

**Table S5. Spatial clustering analysis**

| **Domain** | **Variants #** | **p-value** |
| --- | --- | --- |
| N-terminus | 2 | 0.02 |
| PHMcc | 4 | 0.63 |
| linker | 3 | 0.20 |
| PALcc | 7 | 0.12 |
| C-terminus | 0 | 0.94 |

The spatial clustering of a set of variants is described by calculating the geometric mean distance between all pairs of variants and normalized to the gene's cDNA length (2,922 bp, isoform P19021-5). An empirical p-value is calculated by randomly-generating 100,000 permutations of the variants and comparing their clustering distance against the clustering of the actual variants. As input, we gave the 16 prioritized variants found in the cohort of persons with pituitary adenomas and reported in Table 2. We then classified the number of variants located in particular domains of the protein (converted to cDNA coordinates) and counted the number of variants appearing in each domain across all the permutations to calculate an empirical p-value comparing the actual number of variants found in each domain to the number of variants in each domain in the 100,000 permutations analysis.

**Table S6. Rare (MAF < 1%) and likely pathogenic *PAM* variants that are significantly associated with diagnoses of tumoral/hyperfunctioning pituitary gland in the UKBB**

| **SNP ID** | **DNA/Protein change** | **Location in protein** | **UKBB MAF (%)** | **CADD_PHRED Score** | **Carriers – No diagnosis** | **Non carriers – No diagnosis** | **Carriers - Diagnosis** | **Non carriers - Diagnosis** | **P-value** | **Diagnosis** |
| --- | --- | --- | --- | --- | --- | --- | --- | --- | --- | --- |
| rs61729214 | p.Val351Met | PHMcc | 0.0095 | 25.00 | 36 | 200300 | 2 | 242 | 1.32E-11 | D352 |
| rs150469595 | p.Ile554Val | PALcc | 0.0813 | 25.20 | 324 | 200201 | 2 | 49 | 8.36E-07 | E220 |
| rs368974389 | c.2746+3A>T | PALcc | 0.0401 | 23.50 | 160 | 200364 | 1 | 50 | 0.0232 | E220 |
| rs150662320 | p.Val597Met | PALcc | 0.0439 | 28.90 | 175 | 200141 | 1 | 50 | 0.0314 | E221 |
| n.a. | p.Pro192Arg | PHMcc | 0.0018 | 23.10 | 6 | 194611 | 1 | 204 | 9.29E-09 | E222 |
| rs145099851 | p.Arg190His | PHMcc | 0.1290 | 19.39 | 516 | 200009 | 1 | 4 | 1.74E-05 | E229 |
| rs772356278 | p.Gly531Cys | PALcc | 0.0002 | 31.00 | 0 | 200519 | 1 | 79 | 3.48E-138 | E237 |
| rs1201940345 | c.2689+34G>A | n.a. | 0.0007 | 15.93 | 2 | 200189 | 1 | 79 | 4.36E-47 | E237 |
| rs61729214 | p.Val351Met | PHMcc | 0.0095 | 25.00 | 37 | 200463 | 1 | 79 | 8.17E-05 | E237 |
| rs770736888 | p.Pro693Leu | PALcc | 0.0160 | 23.40 | 63 | 200459 | 1 | 79 | 0.0029 | E237 |
| n.a. | p.Ser97Arg | PHMcc | 0.0007 | 25.40 | 2 | 200516 | 1 | 57 | 1.95E-64 | E240 |
| COSV57210205 * | p.Leu856Pro | C-ter | 0.0040 | 25.00 | 15 | 200528 | 1 | 57 | 3.23E-13 | E240 |

Variants were annotated using the NC_000005.9(NM_000919.3) reference sequence. MAFs were retrieved from the UKBB database. Only variants with a CADD_PHRED score ≥ 15 and a MAF < 1% are listed. The selected diagnoses are given using the ﻿International Classification of Diseases, Tenth Revision (ICD-10) coding: D352, benign neoplasm of pituitary gland; E220, acromegaly and pituitary gigantism; E221, hyperprolactinemia; E222, syndrome of inappropriate secretion of antidiuretic hormone; E229, hyperfunction of the pituitary gland, unspecified; E237, disorder of pituitary gland (unspecified); E240, pituitary-dependent Cushing disease. n.a., not applicable; *, variant ID reported in the COSMIC database.

**Table S7: Gene damage index (GDI) for genes harboring germline and somatic variants known to predispose to pituitary tumors**

|  | **Gene** | **GDI (Phred homogenized)** | **Mean** | **Median** | **Max** | **Min** | **SD** | **95% CI** | **Upper bound (GDI cutoff)** | **Lower bound** |
| --- | --- | --- | --- | --- | --- | --- | --- | --- | --- | --- |
| Germline | *AIP* | 1.160 | 3.829 | 2.161 | 11.375 | 0.196 | 3.991 | 3.8±2.3 | 6.087 | 1.571 |
|  | *MEN1* | 1.434 |  |  |  |  |  |  |  |  |
|  | *CDKN1B* | 9.581 |  |  |  |  |  |  |  |  |
|  | *PRKAR1A* | 0.219 |  |  |  |  |  |  |  |  |
|  | *GPR101* | 11.375 |  |  |  |  |  |  |  |  |
|  | *CABLES1* | 8.032 |  |  |  |  |  |  |  |  |
|  | *DICER1* | 2.685 |  |  |  |  |  |  |  |  |
|  | *SDHA* | 6.694 |  |  |  |  |  |  |  |  |
|  | *SDHB* | 2.675 |  |  |  |  |  |  |  |  |
|  | *SDHC* | 0.196 |  |  |  |  |  |  |  |  |
|  | *SDHD* | 1.647 |  |  |  |  |  |  |  |  |
|  | *MAX* | 0.248 |  |  |  |  |  |  |  |  |
| Somatic | *GNAS* | 3.644 | 2.828 | 2.434 | 5.593 | 0.391 | 1.620 | 2.8±1.1 | 3.950 | 1.705 |
|  | *PIK3CA* | 4.246 |  |  |  |  |  |  |  |  |
|  | *USP8* | 5.593 |  |  |  |  |  |  |  |  |
|  | *USP48* | 2.337 |  |  |  |  |  |  |  |  |
|  | *BRAF* | 1.627 |  |  |  |  |  |  |  |  |
|  | *SF3B1* | 0.391 |  |  |  |  |  |  |  |  |
|  | *ATRX* | 2.251 |  |  |  |  |  |  |  |  |
|  | *NR3C1* | 2.531 |  |  |  |  |  |  |  |  |
| All |  |  | 3.428 | 2.434 | 11.375 | 0.196 | 3.231 | 3.4±1.4 | **4.844** | 2.012 |
|  | ***PAM*** | **3.979** |  |  |  |  |  |  |  |  |

﻿The GDI score for *PAM* is below the upper limit of the calculated confidence interval (GDI cutoff) above which a gene is considered a false positive. GDI scores were retrieved from <http://pec630.rockefeller.edu:8080/GDI/>.

CI, confidence interval; Max, highest GDI within the subgroup; Min, lowest GDI within the subgroup; SD, standard deviation.

**Table S8. *PAM* variants tested to assess their effect on splicing**

| **DNA change** | **Protein change** | **SNP ID** | **Location in gene** | **Location in protein** | **MAF in our cohort (%)** | **Global Control MAF (%)** | **Highest Control Population MAF (%)** | **# of homozygotes in gnomAD** | **P-value** | ***In silico* prediction** | **Individuals harboring the variant** |
| --- | --- | --- | --- | --- | --- | --- | --- | --- | --- | --- | --- |
| c.92T>A | p.Phe31Tyr | rs114014768 | exon 2 | proregion | 0.63 | 0.8654 | 2.814 (African) | 19 | n.s. | activates cryptic SD | 2 acro, 1 gigantism, 1 pediatric CD |
| c.153T>C | p.Pro51= | rs200231533 | exon 2 | PHMcc | 0.16 | 0.0026 | 0.0827 (South Asian) | 0 | <0.05 | benign | 1 gigantism |
| c.195G>T | p.Gly65= | rs145666758 | exon 2 | PHMcc | 0.16 | 0.1908 | 1.995 (Finnish) | 7 | n.s. | benign | 1 acro |
| c.443-79G>T ^†^ | n.a. | rs75448780 | intron 5 | n.a. | 1.56 | 6.366 | 18.20 (East Asian) | 606 | <0.05 | activates cryptic SA | 5 acro, 2 gigantism, 1 NFPA, 2 pediatric CD |
| c.443-5T>C ^†^ | n.a. | rs112626709 | intron 5 | n.a. | 0.16 | 0.4462 | 1.581 (African) | 4 (African) | n.s. | disrupts canonical SA | 1 acro |
| c.1614-32G>A ^†^ | n.a. | rs74633711 | intron 15 | n.a. | 0.15 | 0.4943 | 1.776 (African) | 7 (African) | n.s. | benign | 1 acro |
| c.1804-5A>T | n.a. | rs79621119 | intron 17 | n.a. | 0.31 | 0.7847 | 2.622 (African) | 20 | n.s. | VUS | 2 pediatric CD |
| c.2145C>A | p.Thr715= | rs34746026 | exon 19 | PALcc | 0.46 | 0.2947 | 1.782 (South Asian) | 3 | n.s. | benign | 2 acro, 1 pediatric CD |
| c.2331+76A>C | n.a. | rs139754655 | intron 20 | n.a. | 0.15 | 1.225 | 4.352 (African) | 35 (African) | <0.05 | activates cryptic SA | 1 PRL |

Nine SNVs (three synonymous, one missense, and five intronic) were functionally evaluated for their effect on splicing. Variants were annotated using the NC_000005.9(NM_000919.3) reference sequence. All variants were observed in heterozygosis. MAFs were retrieved from the gnomAD database. When available, combined exome and genome frequencies were used. “European” excludes the Finnish population; “African” includes African-American; “Latino” includes Admixed American. Individual variant allele frequencies in the study population were compared with the global frequencies reported in gnomAD using the Fisher’s exact test or the chi-square test, as appropriate. For *in silico* predictions, the computational verdict was based on the combined outputs of four softwares (Splice Site Finder, MaxEnt, NNSplice, and GeneSplicer) available in Alamut.

acro, acromegaly; CD, Cushing disease; MAF, minor allele frequency; n.a., not applicable; n.s., not significant; PAs, pituitary adenomas; SA, splice acceptor site; SD, splice donor site; VUS, variant of uncertain significance.

^†^One acromegalic harbors the c.443-79G>T and c.443-5T>C variants in the same allele as well as the c.1614-32G>A variant (phase unknown).

# **Supplemental Materials and Methods**

**CLUSTAL Omega v1.2.4 multiple sequence alignment**

tr|A0A0S2C767|A0A0S2C767_CHLRE ---MAPGRL-----FGLLVAVALAL------------------------QAYA---QLPV 25

sp|Q9W1L5|PAL2_DROME ------------------------------------------------------------ 0

sp|O01404|PHM_DROME MPRISEIAASVGL-LLLIGVIS-------------VDGLVKE--GDYQNSLYQQNLESNS 44

tr|Q9NJI4|Q9NJI4_APLCA ---MC-VRAPM----TRLGV--LAVCCVV-SLVMSPTLS--------ADPTTRVMDEVIE 41

sp|P14925|AMD_RAT ---MA-GRARSGLLLLLLGLLALQSSCLAFRSPLSVFKRFKETTRSFSNECLGTIGPVTP 56

**sp|P19021-5|AMD_HUMAN** ---MA-GRV-----PSLLVLLVFPSSCLAFRSPLS**V**FKR**F**KETTRPFSNECLGTTRP**V**VP 51

tr|A0A2I3SM67|A0A2I3SM67_PANTR ---MA-GRV-----PSLLVLLVFPSSCLAFRSPLSVFKRFKETTRPFSNECLGTTRPVVP 51

tr|A0A0S2C767|A0A0S2C767_CHLRE VVSHSIEVNITVPPFKVDQDDAYICVSALLPPHP-HKLVGIIPHAKQEVVHHILLYGCTE 84

sp|Q9W1L5|PAL2_DROME ------------------------------------------------------------ 0

sp|O01404|PHM_DROME ATGATASFPFLMPNVSPQTPDLYLCTPIKVDPTTTYYIVGFNPNATMNTAHHMLLYGCGE 104

tr|Q9NJI4|Q9NJI4_APLCA ADPTTHSMSLLMRGAKPSQPDAYLCTAYPV-TDLETYIYKFQAQANASTAHHMLLYGCDG 100

sp|P14925|AMD_RAT LDASDFALDIRMPGVTPKESDTYFCMSMRLPVDEEAFVIDFKPRASMDTVHHMLLFGCNM 116

**sp|P19021-5|AMD_HUMAN** IDSSDFALDIRMPGVTPKQSDTYFCMSM**R**IPVDEEAFVIDFKPRA**S**MDTVHHMLLFGCNM 111

tr|A0A2I3SM67|A0A2I3SM67_PANTR IDSSDFALDIRMPGVTPKQSDTYFCMSMRIPVDEEAFVIDFKPRASMDTVHHMLLFGCNM 111

tr|A0A0S2C767|A0A0S2C767_CHLRE PHMASKDGKPVAWRCDMKPV----------CNGPSSTILYGWGRNAPDLRLPEGVGFSVG 134

sp|Q9W1L5|PAL2_DROME ------------------------------------------------------------ 0

sp|O01404|PHM_DROME PGTS-K----TTWNCGEMNRASQEESASPCGPHSNSQIVYAWARDAQKLNLPEGVGFKVG 159

tr|Q9NJI4|Q9NJI4_APLCA PAYSTA----DIWHC-PSVC------------RGQQTILFAWAKNAPPTELPRDVGHRVG 143

sp|P14925|AMD_RAT PSST-G----SYWFCDEGTC------------TDKANILYAWARNAPPTRLPKGVGFRVG 159

**sp|P19021-5|AMD_HUMAN** PSST-G----SYWFCDEGTC------------TDKANILYAWARNAPPTRLPKGVGFRVG 154

tr|A0A2I3SM67|A0A2I3SM67_PANTR PSST-G----SYWFCDEGTC------------TDKANILYAWARNAPPTRLPKGVGFRVG 154

tr|A0A0S2C767|A0A0S2C767_CHLRE ERTGVKYIVAQVHYLKVR----PPDDHSGVTLLLKPHAVPYAAGLVSF-ASWFTIPPGKK 189

sp|Q9W1L5|PAL2_DROME ------------------------------------------------------------ 0

sp|O01404|PHM_DROME KNSPIKYLVLQVHYAHIDKFKDGSTDDSGVFLDYTEEPRKKLAGTLLL-GTDGQIPA-MK 217

tr|Q9NJI4|Q9NJI4_APLCA QRSNVKTLVLQVHYAKG-FVRNESPDHSGIIVHMTDRRPKFVAGIFLMMSTWFQVPPHRE 202

sp|P14925|AMD_RAT GETGSKYFVLQVHYGDISAFRDNHKDCSGVSVHLTRVPQPLIAGMYLMMSVDTVIPPGEK 219

**sp|P19021-5|AMD_HUMAN** GETGSKYFVLQVHYGDISAFRDNNKDCSGVSLHLT**R**L**P**QPLIAGMYLMMSVDTVIPAGEK 214

tr|A0A2I3SM67|A0A2I3SM67_PANTR GETGSKYFVLQVHYGDISAFRDNNKDCSGVSLHLTRLPQPLIAGMYLMMSVDTVIPAGEK 214

tr|A0A0S2C767|A0A0S2C767_CHLRE SHPIVNTCCYKGYEALTMFAVRVHTHGLGRRVFMTRETWNKTGT---EELVSRDPQLPQS 246

sp|Q9W1L5|PAL2_DROME ------------------------------------------------------------ 0

sp|O01404|PHM_DROME TEHLETACEVNEQKVLHPFAYRVHTHGLGKVVSGYRVRTNSDGEQEWLQLGKRDPLTPQM 277

tr|Q9NJI4|Q9NJI4_APLCA SYPVDMSCVYLEQKPMYPFAFRTHAHGLGKVITGYL-YN-----GTYQLIGKGNPQWPQA 256

sp|P14925|AMD_RAT VVNADISCQYK-MYPMHVFAYRVHTHHLGKVVSGYRVRN-----GQWTLIGRQNPQLPQA 273

**sp|P19021-5|AMD_HUMAN** VVNSDISCHYK-NYPMHVFAYRVHTH**H**LGK**V**VSGYRVRN-----GQWTLIGRQSPQLPQA 268

tr|A0A2I3SM67|A0A2I3SM67_PANTR VVNSDISCHYK-NYPMHVFAYRVHTHHLGKVVSGYRVRN-----GQWTLIGRQSPQLPQA 268

tr|A0A0S2C767|A0A0S2C767_CHLRE FVPTTR-HTIWPGDRLTVTCLFDSSSKTAPVNAGGTHNDEMCNMYTMVYGKTPYL---TM 302

sp|Q9W1L5|PAL2_DROME ------------------------------------------------------------ 0

sp|O01404|PHM_DROME FYNTSNTDPIIEGDKIAVRCTMQST-RHRTTKIGPTNEDEMCNFYLMYYVDHGETLNMKF 336

tr|Q9NJI4|Q9NJI4_APLCA FYPVEDVIEVKPGDSLAARCTYDSTHMDQRVGVGATGSDEMCNFYIMYYTDSSVQRPGAE 316

sp|P14925|AMD_RAT FYPVEHPVDVTFGDILAARCVFTGEGRTEATHIGGTSSDEMCNLYIMYYMEAKYALSFMT 333

**sp|P19021-5|AMD_HUMAN** FYPVGHPVDVSFGDLLAARCVFTGEGRTEATHIGGTSSDEMCNLYIMYYMEAKHAVSFMT 328

tr|A0A2I3SM67|A0A2I3SM67_PANTR FYPVEHPVDVSFGDLLAARCVFTGEGRTEATHIGGTSSDEMCNLYIMYYMEAKHAVSFMT 328

tr|A0A0S2C767|A0A0S2C767_CHLRE CDNNVQDI------RDDSPGALPR------------------------------------ 320

sp|Q9W1L5|PAL2_DROME ---------------------------------------------------------MSR 3

sp|O01404|PHM_DROME CFSQGAPYYFWSN-PDSGLHNIPHIEASTL------------------------------ 365

tr|Q9NJI4|Q9NJI4_APLCA CMNDQLPELTGAHFPSSVSQPLPPNPQLEEVASGHHHAH--------------------- 355

sp|P14925|AMD_RAT CTKNVAPDMFRT-IPAEANIPIPVKPDMVMMHGHHKEAENKEKSALMQQPKQGEEEVLEQ 392

**sp|P19021-5|AMD_HUMAN** CTQNVAPDMFRT-IPPEANIPIP**V**KSDMVMMHEHHKETEYKDKIPLLQQPKREEEEVLDQ 387

tr|A0A2I3SM67|A0A2I3SM67_PANTR CTQNVAPDMFRT-IPPEANIPIPVKSDMVMMHEHHKETEYKDKIPLLQQPKREEEVL--- 384

tr|A0A0S2C767|A0A0S2C767_CHLRE ------------------------------------------------------------ 320

sp|Q9W1L5|PAL2_DROME LLFVALLAISLGYVASSSSNH-------------LPAGL-----AMDLGP-----GVNLN 40

sp|O01404|PHM_DROME ------------------------------------------------------------ 365

tr|Q9NJI4|Q9NJI4_APLCA ------------------HQH---------------------------GMSGGQ------ 364

sp|P14925|AMD_RAT GDFYSLLSKLLGERED-VHVHKYNPTEKTESGSDLVAEIANVVQKKDLGRSDAREGAEHE 451

**sp|P19021-5|AMD_HUMAN** GDFYSLLSKLLGEREDVVHVHKYN**P**TEKAESESDLVAEIANVVQKKDLGRSDAREGAEHE 447

tr|A0A2I3SM67|A0A2I3SM67_PANTR ------------------------------------------------------------ 384

tr|A0A0S2C767|A0A0S2C767_CHLRE ------HSTLVVDPMP-------NWRPPAP---------------------AGTP----- 341

sp|Q9W1L5|PAL2_DROME ERFFDQVRALIKRRLQE-----KGLAKPEQPELAMPLTDDDAVALQNQRSYDNVPLPAAS 95

sp|O01404|PHM_DROME ------------------------------------------------------------ 365

tr|Q9NJI4|Q9NJI4_APLCA -G---GVAQVHTTQVHT------T-QTPT----------------TQTPTTQTPTMQEIH 397

sp|P14925|AMD_RAT EW---GNAILVRDRIHRFHQLESTLRPAES----------RAFSFQQ--PGEGPWEPEPS 496

**sp|P19021-5|AMD_HUMAN** -R---GNA**I**LVRDRIHKFHRLVSTLRPPES----------RVFSLQQPPPGEGTWEP**E**HT 493

tr|A0A2I3SM67|A0A2I3SM67_PANTR -----------------------------------------------------------D 385

tr|A0A0S2C767|A0A0S2C767_CHLRE GD---------ALGAEAVGDATSVTTGPDGTLWVLYRASGVWKSDTFDRKEVITR--KEP 390

sp|Q9W1L5|PAL2_DROME VP-TPVLVENWPTEQHSFGQVTAVAVDPQGSPVVFHRAERYWDVNTFNESNIYYLIEYGP 154

sp|O01404|PHM_DROME ------------------------------------------------------------ 365

tr|Q9NJI4|Q9NJI4_APLCA FDHPLQLNTSWPGVELTVGQVGGVSVDQRGNLYVFHRGSRVWNAASFDIDNNFQF-QDSP 456

sp|P14925|AMD_RAT GDFHVEEELDWPGVYLLPGQVSGVALDSKNNLVIFHRGDHVWDGNSFDSKFVYQQRGLGP 556

**sp|P19021-5|AMD_HUMAN** GDFHMEEALDWPGVYLLPGQVSGVALDPKNNLVIFHR**G**DHVWDGN**S**FDSKFVYQQIGL**G**P 553

tr|A0A2I3SM67|A0A2I3SM67_PANTR QDFHMEEALDWPGVYLLPGQVSGVALDPKNNLVIFHRGDHVWDGNSFDSKFVYQQIGLGP 445

tr|A0A0S2C767|A0A0S2C767_CHLRE VPEAVVLNMNPDTGKILARWGADVFYLPHSISVDQYGNVWVVDVGRHQVLKFDS--KGKQ 448

sp|Q9W1L5|PAL2_DROME IKENTIYVLDAKTGAIKSGWGSNMFYMPHGLTIDLHGNYWITDVAMHQAFKFKP-FSNKP 213

sp|O01404|PHM_DROME ------------------------------------------------------------ 365

tr|Q9NJI4|Q9NJI4_APLCA ITEDVVLVTDSTG-HKIRSFGAGRYFLPHGIQVDHKDNIWLTDVALHQVFKIPA-GSDTP 514

sp|P14925|AMD_RAT IEEDTILVIDPNNAEILQSSGKNLFYLPHGLSIDTDGNYWVTDVALHQVFKLDPHSKEGP 616

**sp|P19021-5|AMD_HUMAN** **I**EEDTILVI**D**PNNAAVLQSSGKNLFYLPHGLSIDKDGNYWVTD**V**ALHQVFKLDPNNKEGP 613

tr|A0A2I3SM67|A0A2I3SM67_PANTR IEEDTILVIDPNNAAVLQSSGKNLFYLPHGLSIDKDGNYWVTDVALHQVFKLDPNNKEGP 505

tr|A0A0S2C767|A0A0S2C767_CHLRE LLVVGKDRQPGAGKDKFCKPTQVAVL-RDGSFIVADGYCNSRVVWFDKTGKYIAESGAIK 507

sp|Q9W1L5|PAL2_DROME LLTIGKRFRPGSSVKHLCKPTSIAVA-TTGEFFIADGYCNSRILKFNAAGKLLRTIPQ-- 270

sp|O01404|PHM_DROME ------------------------------------------------------------ 365

tr|Q9NJI4|Q9NJI4_APLCA TLTIGHRFQHGEELTFFCKPTDVAVL-SSGEFFVSDGYCNSRVVKFSADGKVIKAWGEKN 573

sp|P14925|AMD_RAT LLILGRSMQPGSDQNHFCQPTDVAVEPSTGAVFVSDGYCNSRIVQFSPSGKFVTQWGEES 676

**sp|P19021-5|AMD_HUMAN** VLILGRSMQPGSDQNHFCQPTDVAVDP**G**TGAIYVSDGYCNSRIVQFSPSGKFITQWGEES 673

tr|A0A2I3SM67|A0A2I3SM67_PANTR VLILGRSMQPGSDQNHFCQPTDVAVDPGTGAIYVSDGYCNSRIVQFSPSGKFITQWGEES 565

tr|A0A0S2C767|A0A0S2C767_CHLRE ------------AVVHGVLVDECEGLVYVASREGRKVVALDITDTKKRLQLKATYDMAAA 555

sp|Q9W1L5|PAL2_DROME -----PPEFLSLQVPHAITLLEHLDLLCIADRENMRVVCPKAGLISSHGEGEPAATIQEP 325

sp|O01404|PHM_DROME ------------------------------------------------------------ 365

tr|Q9NJI4|Q9NJI4_APLCA LEFGVSPPPGTFDVPHSVTVSEGTGQLCVADRENGRVQCFDLEG-N------FDHLIRHK 626

sp|P14925|AMD_RAT --SGSSPRPGQFSVPHSLALVPHLDQLCVADRENGRIQCFKTDTKE------FVREIKHA 728

**sp|P19021-5|AMD_HUMAN** --SGSSPLPGQFTVPHSLALV**P**LLGQLCVAD**R**ENGRIQCFKTDTKE------FVREIKHS 725

tr|A0A2I3SM67|A0A2I3SM67_PANTR --SGSSPLPGQFTVPHSLALVPPLGQLCVADRENGQIQCFKTDTKE------FVREIKHS 617

tr|A0A0S2C767|A0A0S2C767_CHLRE GHG-QVWALRFGPYGEQLALTWDEGKDAHLVNVRFPTQFWTLPGTAKLSPHDFTLGGAAT 614

sp|Q9W1L5|PAL2_DROME DLGR-VFGV--AS---------------------FGDIVFAVNGPTS----MLPVRGFTI 357

sp|O01404|PHM_DROME ------------------------------------------------------------ 365

tr|Q9NJI4|Q9NJI4_APLCA EFGPRLFAVEVCP---------------------LQGVLYAVNGPAYDGPSDLTVQGFTV 665

sp|P14925|AMD_RAT SFGRNVFAISY-----------------------IPGFLFAVNGKPYFGDQ-EPVQGFVM 764

**sp|P19021-5|AMD_HUMAN**  SFGRNVFAISY-----------------------IPGLLFAVNGKPHFGDQ-EPVQG**F**VM 761

tr|A0A2I3SM67|A0A2I3SM67_PANTR SFGRNVFAISY-----------------------IPGLLFAVNGKPHFGDQ-EPVQGFVM 653

tr|A0A0S2C767|A0A0S2C767_CHLRE ELSGAGDRFFSVYLASVGVACDTKCGA-LHKFVVVPTGFKLPAQWELETAVAVPSKAKDV 673

sp|Q9W1L5|PAL2_DROME ---------------------DPRSETIIGHWGE----FKNPHSMAVS------------ 380

sp|O01404|PHM_DROME ------------------------------------------------------------ 365

tr|Q9NJI4|Q9NJI4_APLCA ---------------------DMSSGQLLESWNIP-QGLRNPHDLAVD------------ 691

sp|P14925|AMD_RAT ---------------------NFSSGEIIDVFKPVRKHFDMPHDIV-------------- 789

**sp|P19021-5|AMD_HUMAN** ---------------------NFSNGEIIDIFKPVRKHFDMPHDIV-------------- 786

tr|A0A2I3SM67|A0A2I3SM67_PANTR ---------------------NFSNGEIIDIFKPVRKHFDMPHDIV-------------- 678

tr|A0A0S2C767|A0A0S2C767_CHLRE KPGDAKELPHGAVAANHTANVVAKAIAK-GVPAPAKPPAGEEEDEGGAMVADDYDEDVED 732

sp|Q9W1L5|PAL2_DROME -VN-GSALYVTEIGTNHQTNRVWKYVLA-------------------------------- 406

sp|O01404|PHM_DROME ------------------------------------------------------------ 365

tr|Q9NJI4|Q9NJI4_APLCA -PTTCGSVYVGE----LNPRVVWKLTRADRSTTPTPH-LHM------------------- 726

sp|P14925|AMD_RAT -ASEDGTVYIGD----AHTNTVWKFTLTEKMEHRSVKKAGI------------------- 825

**sp|P19021-5|AMD_HUMAN** -ASEDGTVYIGD----AHTNTVWKFTLTEKLEHRSVKKAGI------------------- 822

tr|A0A2I3SM67|A0A2I3SM67_PANTR -ASEDGTVYIGD----AHTNTVWKFTLTEKLEHRSVKKAGI------------------- 714

tr|A0A0S2C767|A0A0S2C767_CHLRE DTEDAEDYENTKKVLDEEMEAEEEAMADYEEMITGVRPNTTHDKGELVPLDKERYQVLMQ 792

sp|Q9W1L5|PAL2_DROME ------------------------------------------------------------ 406

sp|O01404|PHM_DROME ------------------------------------------------------------ 365

tr|Q9NJI4|Q9NJI4_APLCA ---DTQIVNNNKKN--Q--------------------------IGQAGPSD----TQ--- 748

sp|P14925|AMD_RAT ---EVQEIKEAEAVVEP--------------------------KVENKPTSSE--LQKMQ 854

**sp|P19021-5|AMD_HUMAN** ---EVQEIKEAEAVVET--------------------------KMENKPTSSE--LQKMQ 851

tr|A0A2I3SM67|A0A2I3SM67_PANTR ---EVQEIKEAEAVVET--------------------------KMENKPTSSE--LQKMQ 743

tr|A0A0S2C767|A0A0S2C767_CHLRE DKKAATKTMGSGWANVLVVVLVLALTVISVYYARATLMHYIQSITVSSSNPGGSGAGIKG 852

sp|Q9W1L5|PAL2_DROME ------------------------------------------------------------ 406

sp|O01404|PHM_DROME ------------------------------------------------------------ 365

tr|Q9NJI4|Q9NJI4_APLCA ------------------------------------------------------------ 748

sp|P14925|AMD_RAT EKQKLSTEPGSGVSVVLITTLLVIPV--LVLLAIVMFIRWKKSRAFGD------H-DR-- 903

**sp|P19021-5|AMD_HUMAN** EKQK**L**IKEPGSGVPVVLITTLLVIPV--VVLLAIAIFIRWKKSRAFGA------DSEH-- 901

tr|A0A2I3SM67|A0A2I3SM67_PANTR EKQKLIKEPGSGVPVVLITTLLVIPV--VVLLAIAIFIRWKKSRAFGA------DSEH-- 793

tr|A0A0S2C767|A0A0S2C767_CHLRE GPSAGASTPAGGGLRKTFGAWASAAEGLLGRFSGRGGAAAAAGLAGTTGAG--------- 903

sp|Q9W1L5|PAL2_DROME ------------------------------------------------------------ 406

sp|O01404|PHM_DROME ------------------------------------------------------------ 365

tr|Q9NJI4|Q9NJI4_APLCA ------------------------------------------------------------ 748

sp|P14925|AMD_RAT -------------------KLESSSGRVLGRFRGKGSGGLNLGNFFASRKGYSRKGFDRV 944

**sp|P19021-5|AMD_HUMAN** -------------------KLETSSGRVLGRFRGKGSGGLNLGNFFASRKGYSRKGFDRL 942

tr|A0A2I3SM67|A0A2I3SM67_PANTR -------------------KLETSSGRVLGRFRGKGSGGLNLGNFFASRKGYSRKGFDRL 834

tr|A0A0S2C767|A0A0S2C767_CHLRE ---GAKRSTAEVEAARERERL----LRSGP--- 926

sp|Q9W1L5|PAL2_DROME --------------------------------- 406

sp|O01404|PHM_DROME --------------------------------- 365

tr|Q9NJI4|Q9NJI4_APLCA --------------------------------- 748

sp|P14925|AMD_RAT STEGSDQEKDEDDGTESEEEYSAPLPKPAPSS- 976

**sp|P19021-5|AMD_HUMAN** STEGSDQEK-EDDGSESEEEYSAPLPALAPSSS 974

tr|A0A2I3SM67|A0A2I3SM67_PANTR STEGSDQEK-EDDGSESEEEYSAPLPALAPSSS 866

The PHM and PAL catalytic cores are underlined underneath the human sequence. All missense human variants reported in the study are given in bold. 15 SNVs identified in our cohort of individuals with pituitary adenomas are given in red with light blue highlight, while seven missense SNVs identified in the UKBB are given in dark blue. APLCA, Aplysia californica; CHLRE, *Chlamydomonas reinhardtii*; DROME, *Drosophila melanogaster*; PANTR, *Pan troglodytes*.

***PAM* PCR products cloned into the pSPL3 minigene vector**

Exons are given in uppercase and introns in lowercase

*PAM*-specific primer sequences are underlined

Tested SNVs are given in red

**PAM exon 2 and 100 bp of flanking intronic sequences**

PCR product: 321 bp

SNVs: c.92T>A, c.153T>C, c.195G>T

caatcatctttaaggtcatagaattcctttctttccccttctcatgttgagtttagattccattatgttcaagaatattgaaattgccctcttttttaaggtttaaagaaactaccagaccattttccaatgaatgtcttggtaccaccagacccgtagttcctattgattcatcagattttgcattggatattcgcatgcctggggttacacctaaacaggtgagaggaattttgtctttcattattcacttgttctggatacaatctataattaaagtaaggaaactgcatttacagctgtaggaacttctttaaacgt

**PAM exon 6 and 150 bp of flanking intronic sequences**

PCR product: 384 bp

SNVs: c.443-79G>T, c.443-5T>C

ttatcagtcaatggtatatttctgttttcatataatataattttatatgttatatgttatttccctttggagttcatagaacagcaatttgagattaactccattttagatgctcatttctaatatattaacttttttgtaaatctttagGTGTTGGATTCAGAGTTGGAGGAGAGACTGGAAGTAAATACTTTGTACTACAGGTACACTATGGGGATATTAGTGCTTTTAGAGgtaagttttgaagtgttggaactaagcaaaacttctagtactatattgtttaaaatacatgcacaaactattatctacatcttaaaaagaattgcccacaccctctgcccactaggggataacagcttttgtttcctttaatttggaact

**PAM exon 16 and 100 bp of flanking intronic sequences**

PCR product: 317 bp

SNVs: c.1614-43A>G, c.1614-32G>A

ttgagctcttttgattttgtatttatgtgaatggtctgcatttctcaatttcattggaatttatgattgtttcatgtcctatttaatgctttttgtttagctcgtttgacagcaagtttgtttaccagcaaataggactcggaccaattgaagaagacactattcttgtcatagatccaaataatgctgcagtactccagtccagtggaaaaaatctgtgagttaaatgacttatgttgttaagacttgtactacatatattgagtataaagtgtatcatagagtcttgaaattcgagtgttttgtataactgcact

**PAM exon 18 and 100 bp of flanking intronic sequences**

PCR product: 411 bp

SNV: c.1804-5A>T

gcttctactccaattgcttgtgaaagtggtcttagtcatgtgggtgtaagtcactaataaccatgaaaagtaggtaaggcttttgttcctttttaaaaagGTGTTCAAACTGGATCCAAACAATAAAGAAGGCCCTGTATTAATCCTGGGAAGGAGCATGCAACCAGGCAGTGACCAGAATCACTTCTGTCAACCCACTGATGTGGCTGTGGATCCAGGCACTGGAGCCATTTATGTATCAGATGGTTACTGCAACAGCAGGATTGTGCAGTTTTCACCAAGTGGAAAGTTCATCACACAGTGGGGAGAAGgtacccaataagactcttaatctccagttgtaattcttagccagtatcactgggaactaaatattggccaacactcacattctttaagctgtaattgtcc

**PAM exon 19 and 100 bp of flanking intronic sequences**

PCR product: 401 bp

SNV: c.2145C>A

catttaatggtttactatcatgaatttagaaacatactagagagtcaaactttgggtgatttttaacattgtgtatctaaggcttttttttgttctgcagAgtcttcagggagcagtcctctgccaggccagttcactgttcctcacagcttggctcttgtgcctcttttgggccaattatgtgtggcagaccgggaaaatggtcggatccagtgttttaaaactgacaccaaagaatttgtgagagagattaagcattcatcatttggaagaaatgtatttgcaatttcatatataccaggtatttcatcttaatatgtttgttgtcttctgtcctactgtactctatccttaaaaattgtgttatcttaatgtgctctttttataaattttctttgcta

**PAM exon 20 and 150 bp of flanking intronic sequences**

PCR product: 416 bp

SNV: c.2331+76A>C

agacactaagtattttatattctacttttgttgccaattgtagggaccaggttgaccctaaatttattctttgcataatatacatggatatattagaagtccattcttacccatattttaaagaaaggttaactggatttgctgttgcagGCTTGCTCTTTGCAGTGAATGGGAAGCCTCATTTTGGGGACCAAGAACCTGTACAAGGATTTGTGATGAACTTTTCCAATGGGGAAATTATAGACATCTTCAAGCCAGTGCGCAAGgtatttacacacattgtctaggtttcaattttcatgagaagaagactaaaatataaatcttggcattcaatctgtacttgaatagctttagaaaaacttttaagaagatgtgataactttcttagcaaggttgatgggagtgttctattt

**PAM exon 21 and 100 bp of flanking intronic sequences**

PCR product: 300 bp

SNV: c.2332-2A>T

tttgttgttttgttgtgggctttgctttgtttttctgtcttttcatgaaggattcaagtaaaggctcaccactctaatgtgtagcttcttattttggcagCACTTTGATATGCCTCATGATATTGTTGCATCTGAAGATGGGACTGTGTACATTGGAGATGCTCATACCAACACCGTGTGGAAGTTCACCTTGACTGAGAgtatggttttcacagtattattgttcacattttccctcagtttgattgcttaaaagcatatgaaacaaaaacatttgcatttccttatctttttactgat
